# Targeting Procrastination Using Psychological Treatments: A Systematic Review and Meta-Analysis

**DOI:** 10.3389/fpsyg.2018.01588

**Published:** 2018-08-30

**Authors:** Alexander Rozental, Sophie Bennett, David Forsström, David D. Ebert, Roz Shafran, Gerhard Andersson, Per Carlbring

**Affiliations:** ^1^Department of Clinical Neuroscience, Karolinska Institutet, Stockholm, Sweden; ^2^Institute of Child Health, University College London, London, United Kingdom; ^3^Department of Public Health Sciences, Stockholm University, Stockholm, Sweden; ^4^Department of Psychology and Sport Science, Friedrich-Alexander Universität Erlangen-Nürnberg, Erlangen, Germany; ^5^Department of Behavioural Sciences and Learning, Linköping University, Linköping, Sweden; ^6^Department of Psychology, Stockholm University, Stockholm, Sweden; ^7^Department of Psychology, University of Southern Denmark, Odense, Denmark

**Keywords:** procrastination, psychological treatments, systematic review, meta-analysis, cognitive behavior therapy

## Abstract

**Background:** Procrastination can be stressful and frustrating, but it seldom causes any major distress. However, for some people, it can become problematic, resulting in anxiety, lowered mood, physical complaints, and decreased well-being. Still, few studies have investigated the benefits of targeting procrastination. In addition, no attempt has previously been made to determine the overall efficacy of providing psychological treatments.

**Methods:** A systematic review and meta-analysis was conducted by searching for eligible records in Scopus, Proquest, and Google Scholar. Only randomized controlled trials comparing psychological treatments for procrastination to an inactive comparator and assessing the outcomes by a self-report measure were included. A random effects model was used to determine the standardized mean difference Hedge's *g* at post-treatment. Furthermore, test for heterogeneity was performed, fail-safe *N* was calculated, and the risk of bias was explored. The study was pre-registered at Prospero: CRD42017069981.

**Results:** A total of 1,639 records were identified, with 12 studies (21 comparisons, *N* = 718) being included in the quantitative synthesis. Overall effect size *g* when comparing treatment to control was 0.34, 95% Confidence Interval [0.11, 0.56], but revealing significant heterogeneity, Q(20) = 46.99, *p* < 0.00, and *I*^2^ = 61.14%, 95% CI [32.83, 84.24]. Conducting a subgroup analysis of three out of four studies using cognitive behavior therapy (CBT) found an effect size *g* of 0.55, 95% CI [0.32, 0.77], and no longer showing any heterogeneity, Q(4) = 3.92, *p* = 0.42, *I*^2^ = 0.00%, 95% CI [0.00, 91.02] (*N* = 236). Risk of publication bias, as assessed by the Egger's test was not significant, z = −1.05, *p* = 0.30, fail-safe *N* was 370 studies, and there was some risk of bias as rated by two independent researchers. In terms of secondary outcomes, the self-report measures were too varied to present an aggregated estimate.

**Conclusions:** Psychological treatments seem to have small benefits on procrastination, but the studies displayed significant between-study variation. Meanwhile, CBT was associated with a moderate benefit, but consisted of only three studies. Recommendations for future research are provided, including the use of more valid and reliable outcomes and a screening interview at intake.

## Introduction

### Rationale

For most people, postponing tasks and commitments until the very last minute is quite a harmless endeavor, causing mere annoyance and a bad conscience at worst. For others, however, the behavior is a constant source of anxiety and distress that can turn into something far more harmful. Procrastination, defined as “to voluntarily delay an intended course of action despite expecting to be worse off for the delay” (Steel, 2007, p. 66), is a universal phenomenon with which most individuals are familiar. Although having been conceptualized as involving different types of delay, i.e., arousal, avoidant, and decisional procrastination, empirical evidence has concluded that it consists of only one single underlying factor defined as dysfunctional delay (Steel, 2010). According to research, up to 20% of the adult population regard themselves as struggling with procrastination in their everyday lives (Ferrari et al., 2007). Among university students that number has been found even higher, with at least 50% reporting severe difficulties completing their curricular activities in certain settings (Day et al., 2000). Although not everyone might have a clinical problem that warrants treatment (Rozental and Carlbring, 2014), studies have revealed significant relationships between self-report measures of procrastination, depression, anxiety, stress, and quality of life, with average correlations being in the moderate range (van Eerde, 2003b; Steel, 2007; Beutel et al., 2016). Moreover, procrastination has been linked to perfectionistic concerns (Sirois et al., 2017), rumination and lowered mood (Flett et al., 2016), and excessive worry and generalized anxiety disorder (Stober and Joormann, 2001). Similarly, a number of investigations on the physical and well-being aspects of procrastination have shown that it can affect the ability to initiate and engage in so-called health behaviors, e.g., medical checkups, diets, and exercise (Sirois et al., 2003; 2007). In addition, even though procrastination does not always lead to lower performance (Chun Chu and Choi, 2005), it is rarely seen as a particularly helpful behavior in the context of work and education, resulting in more stress and tension than what is necessary (Rice et al., 2012). Research has also indicated that procrastination can have a negative impact on academic achievement, such as lower grade point average (Steel, 2007), and that it impedes career and financial success (Nguyen et al., 2013).

Still, despite these adverse consequences, procrastination has received relatively little attention with regard to its treatment (Rozental and Carlbring, 2014). Most of the interventions seem to be derived from a motivational or volitional standpoint, such as self-regulation, implementation intentions, goal-setting techniques, and time management (Klingsieck, 2013). Gollwitzer and Sheeran (2006), for instance, showed in a systematic review and meta-analysis that the average between-group effect size of implementation intentions compared to no intervention at post-treatment in 94 studies was Cohen's of *d* 0.65 (implementation intentions are verbal statements that delineate when and how something should be done, e.g., “When in situation X, I will enact behavior Y”). However, the majority of the studies that were included concerned goal achievement in general rather than procrastination *per se*, making unclear to what extent this is in fact an effective treatment for those struggling to initiate and complete tasks and commitments. Similarly, although time management has been put forward as a promising intervention in at least one study (Van Eerde, 2003a), it has not been used to target procrastination specifically. Overall, few controlled studies exist with regard to the use of different interventions. The only randomized controlled trials that have implemented a motivational or volitional standpoint more directly include self-control (Lopez and Wambach, 1982; Davis, 1984), self-monitoring (Pfister, 2002), and goal-setting (Mühlberger and Traut-Mattausch, 2015; Muñoz-Olano and Hurtado-Parrado, 2017). In these cases, the idea has been to overcome procrastination by providing corrective feedback to the individual with regard to how time is being spent, removing distractions to prevent the pursuit of more immediate gratifications, or to increase motivation by manipulating the time frame of completion by using sub-goals, in line with the theoretical understanding of procrastination (Steel, 2007). However, the effect sizes in these studies range from between-group Hedge's *g*s of −0.40–1.42 at post-treatment when compared to wait-list control, suggesting a large variability in outcomes for procrastination, and sometimes even favoring an inactive comparator rather than an intervention. This makes it unclear what the benefits of these interventions are and points to the need of further research.

Meanwhile, from a clinical perspective, different approaches to targeting procrastination have been proposed in the literature, for example, psychodynamic and psychoanalytic treatments (Ferrari et al., 1995). However, no attempts have been made to evaluate their efficacy. One notable exception is a study of coherence therapy (Rice et al., 2011), a type of psychological treatment that is insight-oriented and focuses on experiential methods. It found a within-group effect size *d* of 0.23 on procrastination, but only had a sample size of 18 participants and lacked an inactive comparator. In contrast, cognitive behavior therapy (CBT) and its associated theoretical modalities, e.g., rational-emotive behavior therapy, has long been regarded as helpful for targeting procrastination by clinicians (Pychyl and Flett, 2012), with one of the first self-help books on the subject conceptualizing it as a result of irrational beliefs (Ellis and Knaus, 1977). A more contemporary theory based on CBT however sees procrastination as the result of schedules of reinforcement, sensitivity to delay, and biases and heuristics (Rozental and Carlbring, 2014). Still, very few studies have explored its efficacy and those that exist are mostly single case trials or uncontrolled group therapies (Schouwenburg et al., 2004; Karas and Spada, 2009). While providing some preliminary evidence for its usefulness, research stemming from a clinical perspective on procrastination has involved too many confounders in order to reliably estimate the outcomes of different psychological treatments. A number of recent attempts have, however, started investigating its impact more thoroughly. For example, Rozental et al. (2015a) provided CBT via the Internet over 10 weeks, randomizing self-referred participants from the general population to receive either guidance from a therapist, only self-help, or wait-list control. The results indicated moderate to large between-group effect sizes *d* of 0.50–0.81 on procrastination for both of the conditions when compared to control at post-treatment. Wang et al. (2017) have also conducted a controlled study, randomly assigning participants from a university setting to eight sessions of group CBT, group acceptance and commitment therapy, or wait-list control. The results were similar at post-treatment, with a between-group effect size *g* of 0.61 for CBT when compared to control. Somewhat surprisingly, however, the group acceptance and commitment therapy condition revealed a mere *g* of 0.05. Another study by Toker and Avci (2015) randomly allocated participants from a university setting to eight sessions of group CBT or wait-list control, obtaining results at post-treatment of *g* 0.93 when compared to control. Thus, it appears that psychological treatments do have an influence on procrastination and that more rigorous randomized controlled trials are being conducted, but given the small number of studies and that findings are a bit mixed, its overall efficacy is still unclear.

The objective of the current study was therefore to undertake a more systematic attempt at understanding the benefits of targeting procrastination, using a very broad definition of psychological treatments to identify studies where some form of intervention has been delivered in a pre-specified and coherent fashion. This was deemed important given that procrastination is a highly prevalent phenomenon that can cause concerns and problems to many of those afflicted, but where treatments up to recently have been implemented with very little evidence of their effects. Hence, in order to evaluate the benefits of providing psychological treatments, the current study aims to conduct the first systematic review and meta-analysis of studies that specifically target procrastination. Moreover, given the connection between procrastination and many aspects of well-being, for instance depression and quality of life (van Eerde, 2003b; Steel, 2007; Beutel et al., 2016), the purpose is also to explore the potential benefits of targeting procrastination on secondary outcomes.

## Materials and methods

### Study designs, participants, treatments, and comparators

The current study was designed to test the efficacy of psychological treatments for procrastination using a systematic review and meta-analysis. The aim was to determine the outcome on self-reported procrastination at post-treatment using the following inclusion and exclusion criteria: (1) any psychological intervention specifically targeting procrastination, (2) any self-report measure assessing procrastination, (3) necessary descriptive statistics for calculating standardized mean differences, Hedge's *g*, e.g., sample sizes, means, and standard deviations at post-treatment, and (4) random assignment of participants to treatment or an inactive comparator, e.g., wait-list control. There were, however, no restrictions with regard to publication year, publication type, sample, recruitment, randomization procedure, use of screening interview, or use of secondary outcomes.

### Systematic review protocol

A protocol for the systematic review and meta-analysis was registered prior to data extraction and statistical analysis using the International Prospective Register of Systematic Reviews, PROSPERO (www.crd.york.ac.uk/PROSPERO). The record was last edited on 13/10/2017: CRD42017069981. Three minor deviations were, however, made from the protocol. Firstly, only studies with inactive comparators were included in order to examine the overall efficacy of psychological interventions for procrastination, instead of any form of comparator, i.e., inactive as well as active, as originally stated. Secondly, Proquest was chosen instead of PsycINFO as one of the three databases for the literature search because of their overlap and the former's increased access to doctoral theses. Thirdly, only outcome at post-treatment was explored as there were too few studies reporting data at follow-up.

### Search strategy

Three databases were used for the literature search: Scopus, Proquest, and Google Scholar, which was performed during the period 30/10/2017-3/11/2017. Searches were made using the following search string for both publication titles and abstracts: [TITLE-ABS-KEY (procrastination) AND TITLE-ABS KEY (treatment) OR TITLE-ABS-KEY (intervention) OR TITLE-ABS-KEY (psychotherapy) OR TITLE-ABS-KEY (group) OR TITLE-ABS-KEY (counseling) OR TITLE-ABS-KEY (experimental) OR TITLE-ABS-KEY (randomiz^*^) OR TITLE-ABS-KEY (aid) OR TITLE-ABS-KEY (help) OR TITLE-ABS-KEY (psychological)]. Given that no restrictions were imposed in terms of publication year or publication type, relevant records were reviewed regardless of when they were published or whether they were published articles, doctoral theses, or conference presentations. In addition, screened records were also checked for relevant references in their respective reference list.

### Data sources, studies sections, and data extraction

The records from the literature search were scrutinized in terms of their titles and abstracts. Relevant studies were then reviewed to look for duplicates and the possibility to retrieve records in full text, followed by an assessment of eligibility using the pre-determined inclusion and exclusion criteria. Records included in the quantitative synthesis were then retrieved, with data being extracted using the descriptive statistics provided in either text or tables: sample sizes, means, and standard deviations for the primary outcome. Studies having more than one primary outcome, or where it was unclear which self-report measure was in fact the main outcome, were discussed by AR and DF, selecting only one estimate for the data analysis in order to prevent a violation of independence (Borenstein et al., 2011). In those cases where more than one treatment was being compared to an inactive comparator, these were extracted and presented separately for each comparison. Secondary outcomes were also reviewed where applicable, however, this was only done for self-report measures of conditions other than procrastination, such as psychiatric disorders or outcomes of self-esteem or perfectionism. Furthermore, an assessment of bias was also performed independently by AR and SB using the guidelines provided by the Cochrane Handbook for Systematic Reviews of Interventions (Higgins and Green, 2011).

### Data analysis

The current study used data extracted from the systematic review to perform a meta-analysis of the standardized mean difference *g* at post-treatment. Between-group effect sizes *g*s with their respective 95% Confidence Interval (CI) were calculated using the difference in means between treatment and control for the primary and secondary outcomes, divided by the pooled standard deviations. Following the cutoffs by Cohen (1988), *g*s 0.20–0.50 are believed to represent a small effect, 0.50–0.80 a medium effect, and >0.80 a large effect. However, in line with the recommendations by Cumming and Finch (2001), effect sizes were also compared to other relevant estimates in the literature, such as the efficacy of CBT for psychiatric disorders. Moreover, test for heterogeneity was performed to investigate potential between-study variation, using the *I*^2^-statistic to assess statistical variation across studies (25, 50, and 75% corresponding to low, medium, and high heterogeneity, respectively), and the Q-statistic to test if this heterogeneity was significant (Borenstein et al., 2011). Given that the *I*^2^-statistic can be estimated imprecisely, particularly when the number of studies are few, 95% CIs were also calculated. Furthermore, a forest plot was produced to present the between-group effect sizes for each study and the overall benefits of psychological treatments for procrastination. Lastly, the risk of publication bias was determined using a funnel plot and the Egger's test (Egger et al., 1997). In addition, a fail-safe *N* was calculated to determine the number of studies with a null result that are necessary to increase the *p*-value for the overall effect size above 0.05, using with the Rosenberg-approach (Rosenberg, 2005). All statistical analyses were made in R, using the metafor package (Viechtbauer, 2010), implementing a random effects model as between-study variation was expected.

## Results

### Study selection and characteristics

**Figure 1:**
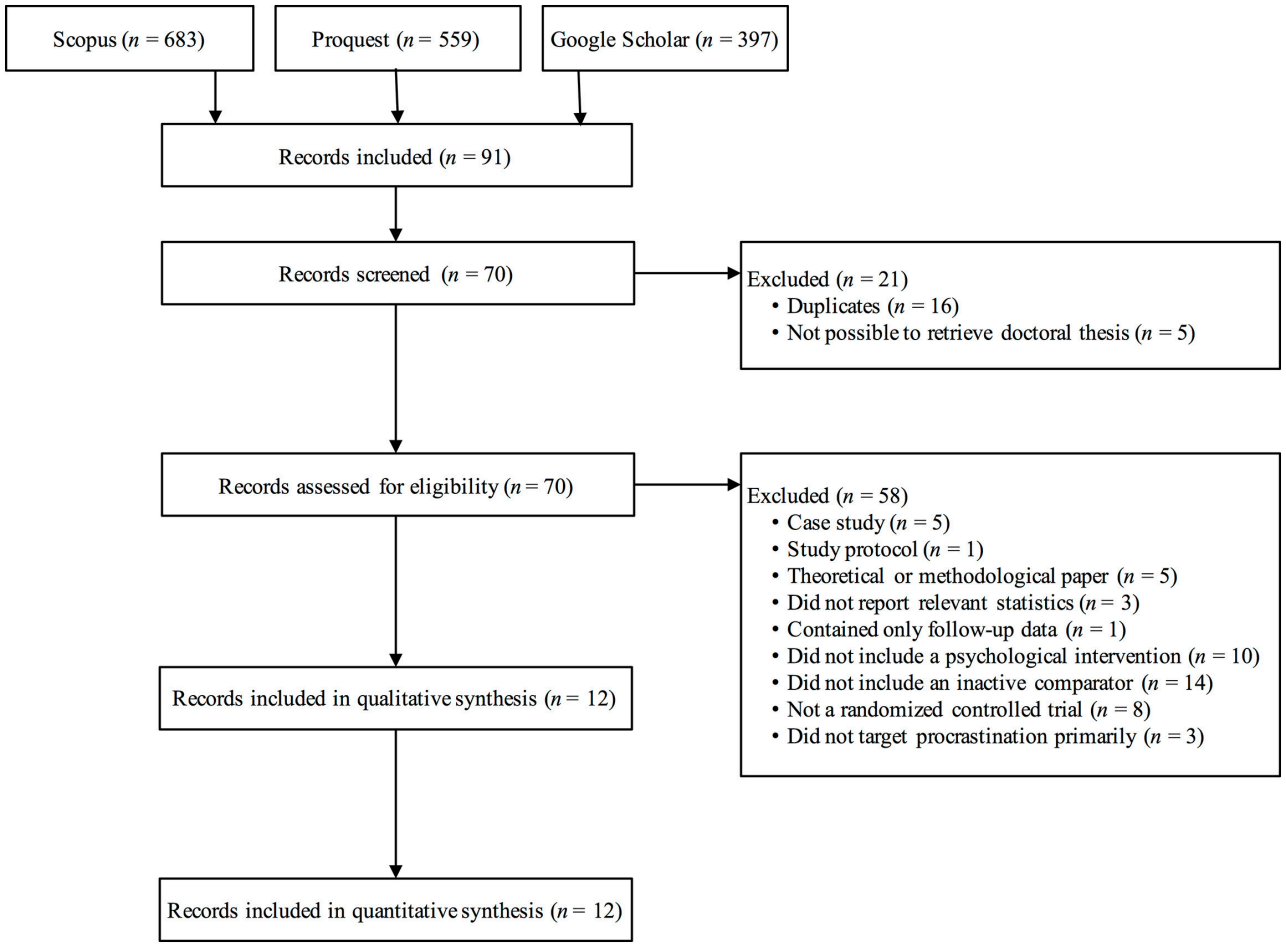
Flow chart of study selection.

The literature search revealed 1,639 records. These were subsequently examined using their titles and abstracts, of which 91 records were found relevant to include. However, 21 records had to be excluded as they were either duplicates (*N* = 16) or doctoral theses that were impossible to retrieve in full text (*N* = 5). Thus, 70 records were assessed for eligibility, with 12 records being included in the quantitative synthesis, totaling 21 comparisons. Hence, 58 records were excluded for a number of reasons, in particular; not employing an inactive comparator, e.g., wait-list control (*N* = 14), not evaluating a psychological intervention, e.g., a computerized monitoring system for course work (*N* = 10), or not being a randomized controlled trial, e.g., counterbalanced AB-BA design (*N* = 8). For a complete flow chart, see Figure 1.

The characteristics of the studies that were included can be seen in Table 1. Four studies were doctoral theses and were eight peer-reviewed articles. Four studies were conducted before 1997, one in 2002, and seven studies during the last 4 years. Further, three studies recruited their samples from the general population, while the rest were either high-school or undergraduate students, but the participants were in all cases self-referrals. There were no clear patterns with regard to the use of primary outcome. However, none was administered in more than three studies, and five assessed academic procrastination. In terms of secondary outcome measures related to other conditions than procrastination, no clear pattern was observed, with none being used in more than one study. As for the type of treatment provided, CBT was most frequently used, delivered individually, in groups, or via the Internet in four studies. Moreover, these studies had the longest treatment durations, ranging from five sessions to 10 weeks. In comparison, the shortest treatment durations were found in a study investigating the use of therapeutic metaphors (Hurley, 1986), which lasted only for a 1-h class, as well as two studies that implemented paradoxical interventions or self-control training, delivered as two 30-min sessions (Lopez and Wambach, 1982; Davis, 1984). In terms of controls, six studies utilized a wait-list control, that is, providing the participants with some form of treatment after the waiting period, while the rest did not specify whether this was the case, i.e., no treatment. Just three studies explicitly described how the randomization procedures were performed, none used a screening interview at intake to assess psychiatric disorders, and the sample sizes in the studies ranged from just six to a maximum of 50 participants.

**Table 1:** Characteristics of the studies included in the meta-analysis.

**Study**	**Publication type**	**Sample and randomization**	**Screening interview**	**Primary outcome**	**Treatment**	**Treatment type**	**Treatment duration**	**Inactive comparator**	**Secondary outcome**
Davis (1984)	Doctoral thesis	High-school students, self-referrals n.a.	No	Procrastination loga (Strong et al., 1979) α = 0.67^*b*^	Paradoxical (19), self-control (18)	Individual, counselors, paradoxical/self-control	2 × 30 min sessions	No treatment (21)	
Eckert et al. (2016)	Peer-reviewed article	General population, self-referrals Random.org	No	General Procrastination Scale –Short version (Klingsieck and Fries, 2012) α = 0.80	Emotion-focused (44)	Web-based, unguided, emotion regulation	2 weeks	Wait-list (39)	Emotion Regulation Skills Questionnaire (Berking and Znoj, 2008)
Hurley (1986)	Doctoral thesis	Undergraduate students, self-referrals n.a.	No	Procrastination loga (Strong et al., 1979) α = 0.67^*b*^	Therapeutic metaphor (13), irrelevant metaphor (13)	Class-based, unguided, metaphorical	1 class × 1 h	No treatment (14)	
Larson (1992)	Doctoral thesis	Undergraduate students, self-referrals n.a.	No	Procrastination Assessment Scale—Student (Solomon and Rothblum, 1984) α = 0.74–85^*b*^	Group CBT (15), individual CBT (8)	Group/individualc, cognitive-behavioral	5 × 120 min sessions (group), 5 × 90 min sessions (individual)	No treatment (15)	The Perfectionism Scale (Hewitt and Dyck, 1986)^*d*^ The Test Anxiety Inventory (Spielberger, 1980)
Lopez and Wambach (1982)	Peer-reviewed article	Undergraduate students, self-referrals n.a.	No	Procrastination loga (Strong et al., 1979) α = 0.67^*b*^	Paradoxical (10), self-control (10)	Individual, counselors, paradoxical/self-control	2 × 30 min sessions	No treatment (12)	
Lukas and Berking (2017)	Peer-reviewed article	General population, self-referrals Random.org	No	General Procrastination Questionnaire (Höcker et al., 2013) α = 0.89	CBM (16)	Smartphone-based, psychologists, cognitive-bias modification	2 group sessions and 2 weeks training	Wait-list (15)	Emotion Regulation Skills Questionnaire (Berking and Znoj, 2008)^*e*^
Mühlberger and Traut-Mattausch (2015)	Peer-reviewed article	Undergraduate students, self-referrals n.a.	No	Academic Procrastination State Inventory (Helmke and Schrader, 2000) α = 0.93	Dyadic coaching (36), group coaching (36)	Group/individual, psychologists in training, coaching	1 × 60 min session	Wait-list (36)	
Muñoz-Olano and Hurtado-Parrado (2017)	Peer-reviewed article	Undergraduate students, self-referrals Random quota selection	No	Procrastination Assessment Scale—Student (Solomon and Rothblum, 1984) α = 0.74–85^*b*^	SMART-goals (16), instructions (16)	Web-based, unguided, goal-setting/checklist	1 month	Wait-list (16)	
Pfister (2002)	Doctoral thesis	Undergraduate students, self-referrals n.a.	No	Procrastination Inventory (Tuckman, 1990) α = 0.77^*b*^	Self-monitoring I (18)^*f*^, self-monitoring II (7)^*f*^	Class-based, unguided, self-monitoring	5 weeks	No treatment I (18)^*f*^, no treatment II (6)^*f*^	
Rozental et al. (2015a)	Peer-reviewed article	General population, self-referrals Random.org	No	Pure Procrastination Scale (Steel, 2010)^*g*^ α = 0.78	Guided ICBT (50), unguided ICBT (50)	Web-based, guided/unguided, cognitive-behavioral	10 weeks	Wait-list (50)	Montgomery Åsberg Depression Rating Scale—Self-report version (Svanborg and Åsberg, 2001) Generalized Anixety Disorder-−7 Items (Spitzer et al., 2006) Quality of Life Inventory (Frisch et al., 1992)
Toker and Avci (2015)	Peer-reviewed article	Undergraduate students, self-referrals n.a.	No	Academic Procrastination Scale (Çakici, 2003) α = 0.92	Group CBT (13)	Groupc, cognitive-behavioral	8 × 90 min sessions	No treatment (13)	State-Trait Anxiety Inventory (Spielberger et al., 1970)^*h*^ Beck Depression Inventory (Beck et al., 1961)^*h*^
Wang et al. (2017)	Peer-reviewed article	Undergraduate students, self-referrals n.a.	No	Academic Procrastination Scale (Milgram et al., 1998) α = 0.83	Group ACT (26), group CBT(26)	Group, psychologists in training, acceptance and commitment therapy/cognitive-behavioral	8 × 180 min sessions	Wait-list (27)	Rosenberg Self-Esteem Scale (Rosenberg, 1965)

*n.a., not available; CBT, cognitive behavior therapy; CBM, cognitive bias modification; ICBT, internet-based cognitive behavior therapy; ACT, acceptance and commitment therapy*.

*^a^ Sum of the Procrastination Behavior Scale*.

b *Internal consistency for the present study not reported*.

*^c^ No information on what therapist or counselors administered the treatment*.

d *Modified version of the Dysfunctional Attitude Scale*.

e *Only used for a mediation analysis*.

f *Study reported statistics divided by university sample, hence two treatments and two comparators*.

g *Study also included the Irrational Procrastination Scale*.

h *Not reported*.

### Synthesized findings

#### Effects on procrastination

**Figure 2:**
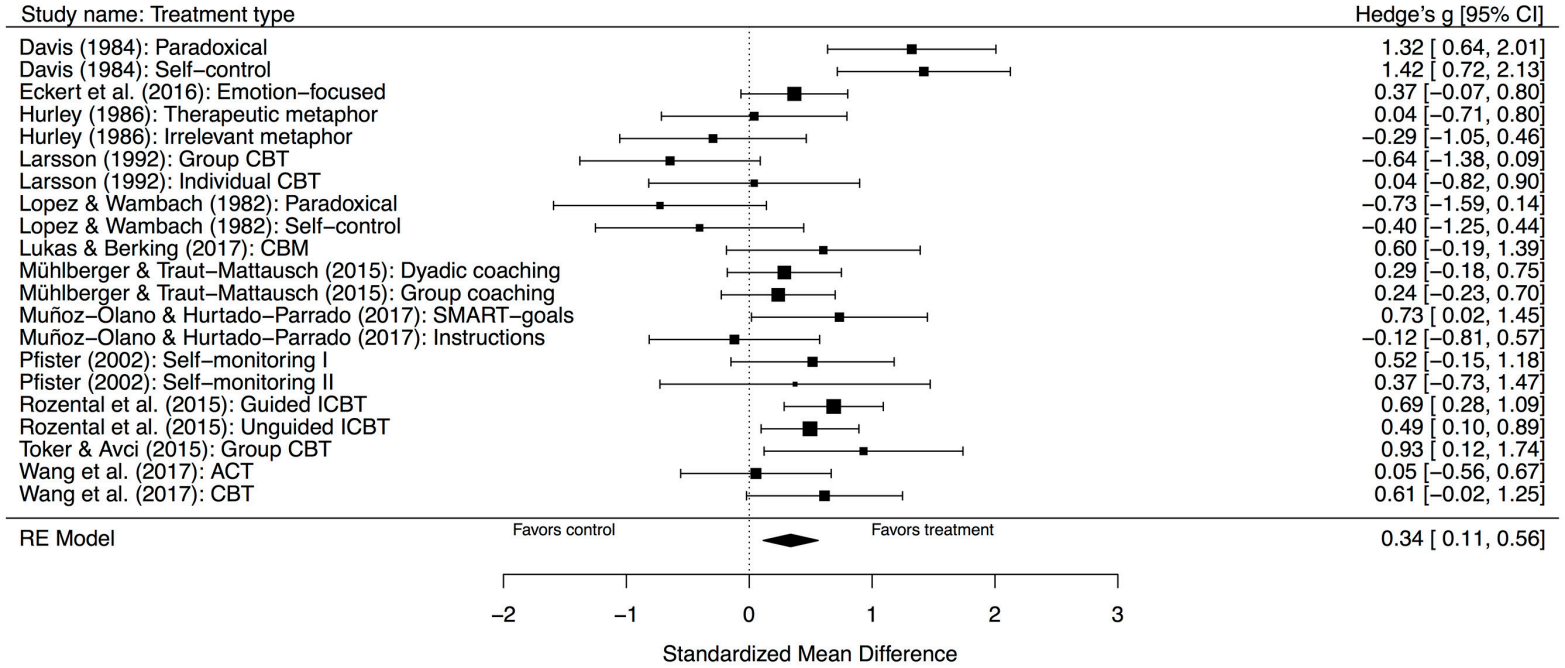
Forest plot displaying the effect sizes of studies comparing psychological treatments with inactive comparators. RE, random effects; CI, confidence interval.

Psychological treatments for procrastination were compared to controls in 12 studies with 21 comparisons and 718 participants (443 in treatment and 275 in control). The standardized mean difference *g* at post-treatment was 0.34, 95% CI [0.11, 0.56], *p* < 0.00, representing a small between-group effect size. However, the test for heterogeneity revealed significant moderate to large between-study variation, Q(20) = 46.99, *p* < 0.00, and *I*^2^ = 61.14%, 95% CI [32.83, 84.24]. Thus, there was greater heterogeneity in the results compared to what would be expected from sampling error alone. A forest plot of this analysis can be seen in Figure 2.

A subgroup analysis of only those four studies that used CBT was also performed, seeing as this was the only type of treatment provided in more than two cases and which had longer treatment durations. This included seven comparisons, totaling 274 participants (175 in treatment and 99 in control). The standardized mean difference *g* at post-treatment was almost the same, 0.35, 95% CI [−0.01, 0.70], *p* = 0.06, although, not significant. The test for heterogeneity was, however, still significant, Q(6) = 13.90, *p* = 0.03, *I*^2^ = 59.62%, 95% CI [0.00, 93.44].

**Figure 3:**
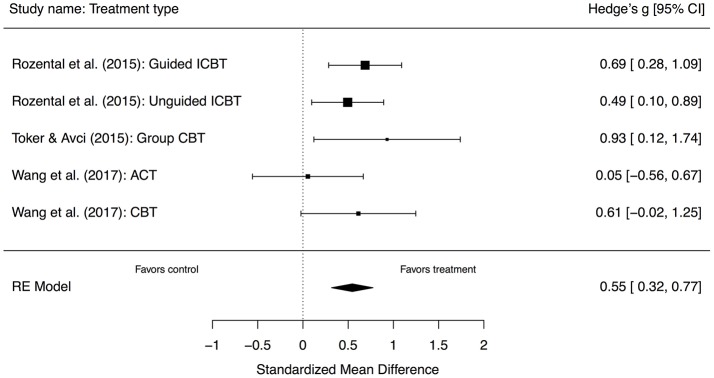
Forest plot displaying the effect sizes of studies comparing cognitive behavior therapy with inactive comparators. RE, random effects; CI, confidence interval.

Given the large difference between the studies, especially with regard to Larson (1992), which in fact favored control when compared to group CBT, *g* of −0.64, 95% CI [−1.38, 0.09], and had very small sample sizes (*n*s varying from 8 to 15), a separate subgroup analysis was completed with this study removed, totaling 236 participants (152 in treatment and 84 in control). The ensuing results revealed a standardized mean difference *g* at post-treatment of 0.55, 95% CI [0.32, 0.77], *p* < 0.00, representing a moderate between-group effect size. Further, the test for heterogeneity was not significant, Q(4) = 3.92, *p* = 0.42, I^2^ = 0.00%, 95% CI [0.00, 91.02], implying that there was no longer a between-study variation of effects. A forest plot of this analysis can be seen in Figure 3.

**Figure 4:**
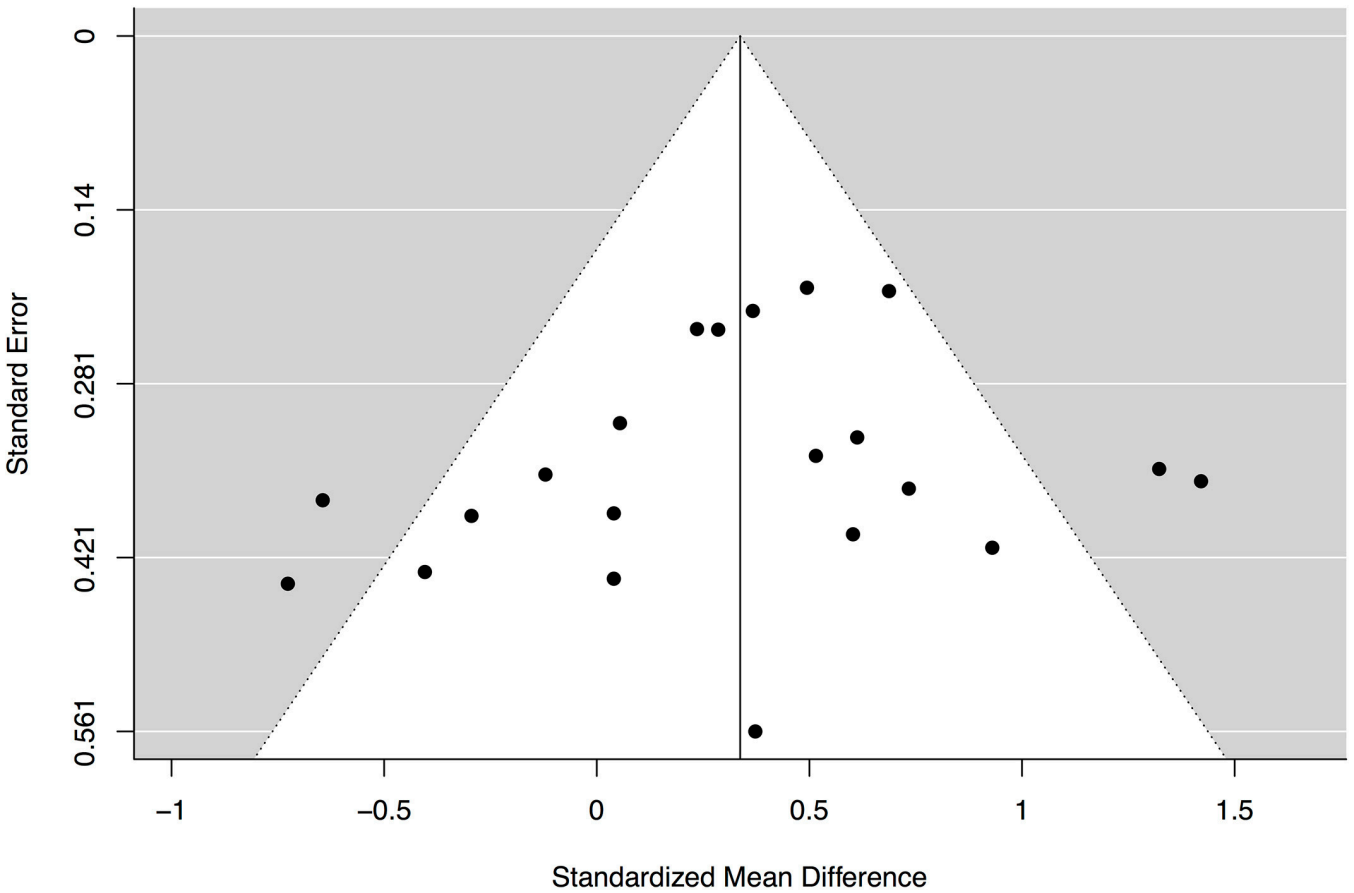
Funnel plot assessing the risk of publication bias.

Lastly, a funnel plot was produced to investigate the risk of publication bias, as seen in Figure 4. Performing a visual inspection suggested some funnel plot asymmetry. However, the Egger's test was not significant, z = −1.05, *p* = 0.30, indicating that the observation of asymmetry was not supported and that there may not be a risk of publication bias. In addition, the fail-safe *N* indicated that an additional 370 studies with a null result is required in order to make the overall effect size non-significant.

#### Effects on secondary outcome measures

Given the large variation of secondary outcomes that assessed conditions other than procrastination in the included studies, a synthesis of the results was not feasible. However, the findings from each of the comparisons at post-treatment are nonetheless important for future reference, which is why these are examined and presented individually. The study by Eckert et al. (2016), for example, used the Emotion Regulation Skills Questionnaire (Berking and Znoj, 2008), comprised of nine subscales on emotion regulation skills, e.g., acceptance of aversive emotions. The between-group effect sizes *g*s ranged from −0.59 to 0.48, 95% CIs [−1.02–0.91], however, only the subscales awareness, sensation, and clarity were significant and favoring treatment.

The study by Larson (1992) examined the effects on perfectionism using the Perfectionism Scale (Hewitt and Dyck, 1986), which favored the control compared to the two treatments that were provided, *g*s −0.17, 95% CI [−0.88, 0.55] for group CBT, and −0.29, 95% CI [−1.14, 0.58] for individual CBT. In contrast, using a self-report measures of test anxiety, the Test Anxiety Inventory (Spielberger, 1980), there were some benefits for those participants receiving treatment, *g*s 0.23, 95% CI [−0.50, 0.94] for group CBT, and 0.34, 95% CI [−0.54, 1.19] for individual CBT, although, none of these comparisons were significant.

The study by Rozental et al. (2015a) investigated the impact on depression, anxiety, and quality of life using the Montgomery Åsberg Depression Rating Scale—Self-report version (MADRS-S; Svanborg and Åsberg, 2001), the Generalized Anixety Disorder−7 Items (GAD-7; Spitzer et al., 2006), and the Quality of Life Inventory (QOLI; Frisch et al., 1992). This is presently the only study that has assessed the effects of a psychological treatments of procrastination on psychiatric disorders and well-being, as compared to an inactive comparator. For depression: *g* of 0.41, 95% CI [0.01, 0.81], for guided ICBT, and 0.10, 95% CI [-0.29, 0.49], for unguided ICBT, however, only the former was significant. For anxiety: *g* of 0.23, 95% CI [−0.16, 0.62], for guided ICBT, and 0.05, 95% CI [−0.34, 0.44], for unguided ICBT, none being significant. For quality of life: *g* of 0.40, 95% CI [0.00, 0.80], for guided ICBT, and 0.22, 95% CI [−0.17, 0.61], for unguided ICBT, with only the former being significant.

Furthermore, the study by Wang et al. (2017) determined the effects on self-esteem using the Rosenberg Self-Esteem Scale (Rosenberg, 1965), with all results in favor of control, *g* of −0.76, 95% CI [−1.38, −0.12] for group ACT, and −1.14, 95% CI [−1.78, −0.45] for group CBT, both being signficant.

### Risk of bias

An assessment of bias was conducted independently by AR and SB using the guidelines provided by the Cochrane Handbook for Systematic Reviews of Interventions (Higgins and Green, 2011). The results varied between studies, but some risk of bias was evident in all of the cases. Most frequently endorsed were the items “Blinding of participants and personnel” and “Blinding of outcome assessment,” where 92% of the studies were found to have a high risk of bias. In comparison, only one study (8%) was rated as having a high risk of bias for “Selective reporting,” and only study (8%) exhibited “Other sources of bias,” which was related to difficulties adhering to a full randomization procedure. A complete overview of the risk of bias assessment can be seen in Table 2.

**Table 2:**
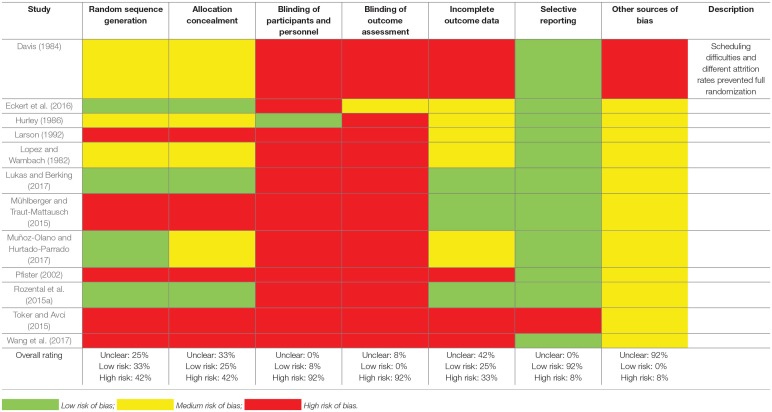
Risk of bias assessment of the studies included in the meta-analysis.

*Low risk of bias; Medium risk of bias; High risk of bias*.

## Discussion

The current study is the first systematic review and meta-analysis of psychological treatments for procrastination. The results revealed that despite being a highly prevalent condition, especially among high-school and university students (Ferrari et al., 2007; Steel and Klingsieck, 2016), very few studies exist with regard to its treatment. This is made clear by the fact that only 12 out of 1,694 records were obtained and found eligible for a meta-analysis, suggesting that there is great need for further research and more randomized controlled trials, preferably cases comparing treatment to an inactive comparator. Still, based on 21 comparisons and 718 participants, the standardized mean difference *g* at post-treatment was 0.34, indicating a small but nonetheless significant between-group effect size when compared to control, giving some credence to the use of psychological treatments for procrastination. However, there was also large heterogeneity, revealing a between-study variation of effects that is probably related to differences among the studies. In addition, five comparisons were found to have negative effect sizes, thus favoring control over treatment. This is not unexpected given that many of the records were doctoral theses, sample sizes were small, and that the type of treatment provided and their durations varied considerably. The overall effect size should thus be interpreted cautiously and underlines the importance of additional high-quality and adequately powered studies that employ more rigorous designs. In particular, future randomized controlled trials should make an effort to prevent the risk of bias, which was found to be high in most of the studies. Especially, blinding of the outcome assessment is concerning, which is important and usually does not require more than the implementation of a code key that is not deciphered until it is time to report the results. In addition, other measures to prevent bias could also be improved, most notably, enforcing proper randomization procedures and reporting all of the primary and secondary outcomes that are included.

As for the additional benefits of targeting procrastination with psychological treatments, the variation in secondary outcome measures made it impossible to aggregate the results. Still, there were small to moderate effects for emotion regulation, test anxiety, depression, anxiety, and quality of life, favoring treatment. This might imply that alleviating procrastination could be helpful for other conditions as well, similarly to what has been proposed for perfectionism (Egan et al., 2011). However, more research is needed to confirm these findings, especially since the number of studies reporting these estimates were quite small and not always significant. In addition, the results seemed to go in an opposite direction in two studies exploring the effects for perfectionism and self-esteem, i.e., favoring control, although this should be interpreted cautiously and might just represent an artifact of the self-report measures being used or the random variation in the samples included.

In terms of a subgroup analysis of three out of four studies that used CBT, this revealed that heterogeneity was no longer evident, thereby implying more robust results. In addition, this showed greater benefits compared to control, with a standardized mean difference *g* at post-treatment of 0.55, implying a moderate and significant between-group effect size. However, this was based on only three studies and five comparisons with 236 participants, and despite no longer displaying significant heterogeneity, the 95% CI for the *I*^2^-statistic was still very wide [0.00, 93.44], suggesting this finding should be interpreted cautiously. Moreover, when including the study by Larson (1992), the effect sized dropped markedly to just g of 0.35, making these results tentative at best and warranting further research when more randomized controlled trials become available. Still, this finding is more in line with what is obtained in systematic reviews and meta-analyses of CBT for psychiatric disorders, but with a somewhat lower effect. For instance, Cuijpers et al. (2013) found a *g* of 0.71 for adult depression when compared to control (*g* of 0.53 adjusted for publication bias). Likewise, when reviewing computerized CBT, Andrews et al. (2010) obtained a *g* of 0.88 for both depression and anxiety disorders (panic disorder, social anxiety disorder, and generalized anxiety disorder), in comparison to control. However, investigating the results of the current study with a systematic review and meta-analysis of a similar non-psychiatric disorder would probably be more informative, e.g., perfectionism. Regrettably, the only case that currently exist reported within- rather than between-group effect sizes (Lloyd et al., 2015), making it impossible to infer any similarities or differences in benefits. Looking more closely at specific examples on the other hand, the between-group effect sizes for ICBT for perfectionism when compared to wait-list control ranges between *d* of 0.68 to 1.04 (Rozental et al., 2017; Shafran et al., 2017), indicating higher estimates than the overall benefits obtained in the current study.

Whether CBT should be regarded as the most efficacious type of psychological treatment for procrastination is unclear given the few cases of other theoretical modalities included in the systematic review and meta-analysis. In addition, it should be noted that several of the other studies that were explored used specific interventions that are often employed as part of CBT, such as self-monitoring, and goal setting techniques. Hence, it is not known if other psychological treatments that were not assessed in the current study might also be beneficial. Psychodynamic as well as psychoanalytic approaches to procrastination have for instance been discussed (Ferrari et al., 1995), primarily with regard to the concept of ego defense, i.e., avoiding task completion because failure or success can be threatening to one's self-concept, the influence of attachment styles, and parental control. However, if and how these perspectives can be translated into a clearly defined psychological treatment for procrastination is still unclear, and not a single study was found to have examined their effects. Meanwhile, CBT seems to fit quite well-theoretically with the present understanding of procrastination. Steel (2007) reviewed the literature on the topic in a meta-analysis, proposing that four variables can be used to explain why individuals procrastinate: the value of completing an intended course of action, the expectation to achieve that value, the timing of that value, and sensitivity to delay. Targeting these aspects could therefore be perceived as important, and CBT usually provides corresponding interventions, e.g., value-directed behavior and goal-setting (value), modeling and behavioral experiments (expectancy), sub-goals and behavioral activation (timing), as well as stimulus control and implementation intentions (sensitivity to delay; Rozental and Carlbring, 2014). The only study that has explicitly developed its treatment in accordance with these variables is performed by Rozental et al. (2015a), warranting further exploration and replication by an independent group of researchers. Moreover, it would be interesting from a conceptual point of view to examine whether some of these aspects are more important than others to target, perhaps by dismantling behavioral and cognitive interventions.

Additional studies of psychological treatments for procrastination do of course exist. Lately, Gieselmann and Pietrowsky (2016), Glick and Orsillo (2015), and Hafner et al. (2014) have all conducted randomized controlled trials, administering either implementation intentions, time management, or acceptance-based behavioral interventions. Although indicating some benefits, the treatment durations were very short. Also, because no inactive comparator was not used in any of the cases, distinguishing the impact of the specific treatments is difficult, which is why they were not included in the current study. In addition, Rozental et al. (2018) randomly assigned participants to either unguided Internet-based CBT or group CBT in an 8-weeks or four-session treatment, obtaining promising results, but again without an inactive comparator. However, this study is the only one implementing a screening interview at intake, the MINI-International Neuropsychiatric Interview (MINI; Sheehan et al., 1998), suggesting that 19.6% fulfilled the criteria for an anxiety disorder. It is also the first to evaluate treatment for procrastination in the context of routine care (a student health center). Despite some limitations, these more recent attempts are encouraging, and the systematic review and meta-analysis shows that more than half of the included studies were in fact conducted during the last 4 years, possibly indicating an increased interest among researchers in finding effective psychological treatments for procrastination.

Based on the findings in the current study, some recommendations for future research can be made. First, the systematic use of a screening interview at intake is highly advised, e.g., the MINI (Sheehan et al., 1998). This should help clarify the relationship between procrastination and psychiatric disorders, which, at present, has almost exclusively been derived from correlations of different self-report measures (c.f., Beutel et al., 2016). Second, the distribution of commonly used secondary outcomes is recommended in order to explore the impact of treatment on other conditions, e.g., the MADRS-S (Svanborg and Åsberg, 2001), or the PHQ-9 (Löwe et al., 2004) for depression, the GAD-7 (Spitzer et al., 2006) for anxiety, and the QOLI (Frisch et al., 1992), or the Brunnsviken Brief Quality of Life Scale (Lindner et al., 2016), for quality of life and general well-being. Third, given that studies have almost exclusively assessed the benefits of treating procrastination at post-treatment, long-term effects are relatively unexplored and need to be adressed further to determine if outcomes are maintained over time. Lastly, and most important, although revealing a significant between-group effect size *g* of 0.34 when compared to control, the benefits of psychological treatments for procrastination must be seen as quite modest. Future studies should therefore try to improve the results by providing an adequate dose of the interventions, such as admininstering psychological treatments for at least eight sessions or weeks. This is in line with what is implemented for other similar conditions like perfectionism (Egan et al., 2011), and seems reasonable with regard to the often reccurrent and trait-like behavioral pattern that characterizes many cases of procrastination. Moreover, it is also possible that individuals undergoing treatment for procrastination needs more support to implement the interventions that are included than many psychiatric disorders. For instance, Rozental et al. (2015b) found that many participants struggled to keep up with the treatment they were receving, which became yet another task on their long list of postponed to-dos. One way of overcoming this issue might be to use a therapist-led group setting in order to increase accountability, help those not on track, and create naturally occurring social reinforcers to treatment adherence. Some evidence of this was provided by Rozental et al. (2018), in which participants in group treatment fared better in the long-run than those only receiving an unguided intervention format, who actually deteriorated somewhat in their procrastination after treatment completion. In addition, given the widespread use of smartphones, adding notification systems and components of self-monitoring via applications seem promising in the treatment of procrastination. Such measures should be able to overcome some of the problems associated with interventions so far, such as promoting the use of implementation intentions, sub-goals, and tracking change.

### Limitations

The current study and its results need to be interpreted in relation to a number of limitations. First, efforts were made to find as many studies as possible for the systematic review and meta-analysis. Three databases were used for the literature search, and screened records were also checked manually in terms of their reference lists. However, given that only AR conducted the search, some studies might have been missed out. In addition, although some doctoral theses were accessible via Proquest, others were impossible to retrieve in full text. These may have been obtainable via post and could be interesting to include in the future to see if they affect the overall results. However, given the small sample sizes and high risk of bias in many of these doctoral theses, this assumption seems unlikely. Of greater importance is the so-called gray literature, which includes unpublished manuscripts and conference abstracts that are often not attainable through databases. For instance, presentations at the Biennial Procrastination Research Conference and direct contact with renowned researchers in the field may have revealed additional records, but this was not done in the current study and should be seen as a potential limitation. Moreover, as with all systematic reviews and meta-analyses, there is always a risk of a file-drawer effect, i.e., non-significant findings not being published and thereby affecting the results of the quantitative synthesis. This is particularly relevant given that only one of the included studies was pre-registered as a clinical trial (Rozental et al., 2015a). It is therefore essential that future research on psychological treatments for procrastination is being registered, such as using ClinicalTrials.gov. However, the file-drawer effect was in fact explored using the Egger's test, which was not found to be significant, and the fail-safe *N*, which indicated that more than three-hundred studies with s with a null result would be required to make the overall effect size non-significant. Overall, this suggest that the risk of a file-drawer effect was quite low.

Second, only studies with an inactive comparator were included in the systematic review and meta-analysis. This was done to determine the overall effect of providing psychological treatments for procrastination, something that is not feasible with another form of control. However, as a consequence, a total of 14 studies were excluded from the quantitative synthesis, some of which might have been informative with regard to their benefits for treating procrastination. Furthermore, it is possible that a number of these records were of higher quality and exhibited less risk of bias than those included, possibly affecting the overall effects. Still, given that studies with different comparators are not recommended to include in the same meta-analysis (Borenstein et al., 2011), this was not deemed feasible but could be explored separately in the future.

Third, investigating the results of providing psychological treatments for procrastination is bound to introduce some heterogeneity because of its very broad inclusion criterion. In the current study, any psychological intervention specifically targeting procrastination could be included if found eligible, which means that some were probably more theoretically and methodologically sensible than others. It is, for instance, unclear why the use of a therapeutic metaphor would be enough for treating procrastination, as in the case of Hurley (1986). However, as the research field progresses with additional randomized controlled trials, systematic reviews and meta-analyses should use more restrictive criteria.

Fourth, although the sub-group analysis of only those studies using CBT revealed moderate benefits, *g* of 0.55, one needs to be careful when interpreting this finding as one quarter of the records were omitted (Larson, 1992). This study can be seen as an outlier in terms of its results and small sample size, but excluding it from the quantitative synthesis may also overestimate the results, which was in fact only *g* of 0.35 when it was included. Additional randomized controlled trials are therefore needed before any conclusive evidence on the efficacy of CBT for procrastination can be determined.

Lastly, the use of self-report measures varied greatly between the included studies, some being relatively well-known and administered for decades in relation to procrastination, others being quite unexplored from a psychometric perspective. This could have made an impact on the findings in the current study, particularly if some are less susceptible to change, which would constrain the difference between participants in treatment and control and decrease the between-group effect size. Also, given that some of the primary outcomes were more related to academic rather than general procrastination, the benefits of receiving treatment might not be possible to aggregate. Future studies should therefore use more validated and reliable self-report measures that correspond better to the sample that is being explored.

## Conclusions

The current study is the first systematic review and meta-analysis investigating the benefits of providing psychological treatments for procrastination. The overall effect was small but significant at post-treatment when compared to no treatment. However, due to heterogeneity, the results should be interpreted cautiously. A sub-group analysis of only three out of four studies using CBT was, on the other hand, not heterogeneous and found a medium-sized overall effect favoring treatment. Given the high risk of bias and poor quality in many studies, additional randomized controlled trials are needed, preferably adhering to some of the recommendations provided in the current study. Nevertheless, in line with the ideas proposed by Cumming (2014), the current study should fit well with the concept of “meta-analytic thinking” (p. 23), in which the findings can inform future studies in their research planning, e.g., providing more accurate estimates for calculating statistical power, increasing the accuracy in parameter estimation used for 95% CIs, and, most importantly, accumulating the evidence for a specific issue in psychology that can be replicated and assessed in relation to prior evidence.

## Author contributions

The authors contributed to the current study as follows: AR conducted the systematic review, meta-analysis, risk of bias assessment, and drafted the manuscript. SB did an independent risk of bias assessment and provided feedback on the manuscript. DF outlined the inclusion and exclusion criteria and provided feedback on the manuscript. DE, RS, and GA provided statistical support and feedback on the manuscript, and PC supervised AR and provided feedback on the manuscript.

### Conflict of interest statement

The authors declare that the research was conducted in the absence of any commercial or financial relationships that could be construed as a potential conflict of interest.
